# Transcriptome Analysis and Cell Morphology of *Vitis rupestris* Cells to Botryosphaeria Dieback Pathogen *Diplodia seriata*

**DOI:** 10.3390/genes12020179

**Published:** 2021-01-27

**Authors:** Liang Zhao, Shuangmei You, Hui Zou, Xin Guan

**Affiliations:** 1 College of Horticulture and Landscape Architecture, Southwest University, Chongqing 400716, China; zhao_liang@yeah.net (L.Z.); shuangmeiyou@163.com (S.Y.); zouhuiht@126.com (H.Z.); 2 Key Laboratory of Horticulture Science for Southern Mountainous Regions, Ministry of Education, Chongqing 400716, China

**Keywords:** Botryosphaeria dieback, *Diplodia seriata*, *Vitis rupestris*, defense response, RNA-seq

## Abstract

*Diplodia seriata*, one of the major causal agents of Botryosphaeria dieback, spreads worldwide, causing cankers, leaf spots and fruit black rot in grapevine. *Vitis rupestris* is an American wild grapevine widely used for resistance and rootstock breeding and was found to be highly resistant to Botryosphaeria dieback. The defense responses of *V. rupestris* to *D. seriata* 98.1 were analyzed by RNA-seq in this study. There were 1365 differentially expressed genes (DEGs) annotated with Gene Ontology (GO) and enriched by the Kyoto Encyclopedia of Genes and Genomes (KEGG) database. The DEGs could be allocated to the flavonoid biosynthesis pathway and the plant–pathogen interaction pathway. Among them, 53 DEGs were transcription factors (TFs). The expression levels of 12 genes were further verified by real-time quantitative reverse transcription polymerase chain reaction (qRT-PCR). The aggregation of proteins on the plasma membrane, formation variations in the cytoskeleton and plasmodesmata and hormone regulations revealed a declined physiological status in *V. rupestris* suspension cells after incubation with the culture filtrates of *D. seriata* 98.1. This study provides insights into the molecular mechanisms in grapevine cells’ response to *D. seriata* 98.1, which will be valuable for the control of Botryosphaeria dieback.

## 1. Introduction

Botryosphaeria dieback, together with Esca, Eutypa dieback and Phomopsis, is the most important complex grapevine trunk disease (GTD) worldwide [1]. The progression from infection to symptom onset is chronic and accumulates over time, resulting in production reduction, economic losses and even vineyard disruption. In the worst case, it leads to the need to replant the entire vineyard long before the normal useful life of the vineyard is reached [1,2,3,4]. Botryosphaeria dieback caused by botryosphaeriaceous fungi is probably the most widespread GTD in the world [5]. In addition to colonization of the vine by pruning wounds, invasion of botryosphaeriaceous fungal agents occurs by airborne inoculum during rainfall in the vegetative growth period [2,6]. In recent decades, many efforts have been made to either protectively optimize the pruning time and technique or use pruning wound protectants; however, no efficient protective or curative measures have yet been found to control this aggressive disease [2]. 

The species within the Botryosphaeriaceae that infect grapevines have been proposed to be classified into three virulence levels, including highly virulent species (*Lasiodiplodia* spp. and *Neofusicoccum* spp.), moderately virulent species *(Botryosphaeria dothidea* and *Diplodia* spp.) and weakly virulent species (*Dothiorella* spp. and *Spencermartinsia viticola*) [2]. Since the pathogens were detected in the wood but never in the leaves of infected plants, it was hypothesized that leaf symptoms might be caused by extracellular compounds of the fungi in the trunk transported by the transpiration stream [7,8]. Fungal filtrates (containing extracellular compounds) have been widely used since the 1980s as a substitute of the infective agent since the productions of host-selective toxins are involved as pathogenicity determinants [9,10,11]. Several toxic factors were found to be synthesized by the species of Botryosphaeriaceae, including mellein, cis, trans-4-hydroxymellein, 4,7-dihydroxymellein, naphthalene enone-related compounds and lipophilic low-molecular weight phytotoxins (exopolysaccharides, peptides or proteins) [7]. It has been demonstrated that extracellular compounds produced by Botryosphaeriaceae species induced defense gene expression in grapevine cells [7,12,13]. Moreover, extracellular proteins produced by *N. parvum* induced more intense early defense responses in *Vitis rupestris* cells than those produced by *Diplodia seriata* [4]. In contrast to *N. parvum*, *D. seriata* is a less aggressive pathogen. Understanding its genetic and molecular basis and identifying key genes responsible for defense may provide valuable insights for GTD control. 

Previous studies showed that a great number of genes are associated with defense responses to grapevine fungal diseases. The extracellular compounds from *D. seriata* induced the expression of glutathione S-transferase (*GST1*), phenylalanine ammonia-lyase (*PAL*), stilbene synthase (*STS*), pathogenesis-related protein 6 (*PR6*), class IV chitinase (*Chit4c*) and pathogenesis-related protein 10 (*PR10*) in *V. vinifera* cv. Chardonnay at 3 days post-inoculation (dpi) but not at 1 dpi [12]. The expression of superoxide dismutase (*SOD*), hypersensitive response (*HSR*), *STS* and *PR* was evaluated in *V. vinifera* subsp. *sylvestris* as early as 12 hours post-inoculation (hpi) [14]. These results showed that some of these genes were not only up-regulated after incubation, but also correlated with cultivar susceptibility to *D. seriata*. 

Subcellular components are involved in defense responses. A target of toxin action by Eutypa dieback is in the plasmalemma, as found in infection of *V. vinifera* cv. Cabernet Sauvignon [15]. Recent advances revealed that cellular connectivity via regulation of plasmodesmata permeability is tightly related to plant defense pathways [16,17]. Plant microtubules (MTs) have been proposed for signaling transduction in plant defense responses [18]. Several studies indicated that MTs not only play the role as the scaffold of the cell, but are also involved in the activation of downstream gene expressions in grapevine cells responding to biotic [19] and abiotic stresses [20]. A *V. rupestris* cell suspension culture expressing GFP-*At*TUB6 was established and used to investigate the function of MTs in processing and decoding stress signals under very early biotic/abiotic stress conditions [21]. Some of the toxic extracellular proteins from *D. seriata* have been identified [7,12], where the *V. rupestris* cells were used and revealed a rapid increase in extracellular pH, superoxide dismutase (ROS) production, cell death and the expression of some defense genes (*VvPR1*, *VvPR6*, *VvPR10.1*, *VvSTS1* and *VvChit4c*) [4]. However, the comprehensive mechanisms at the transcriptional level of Botryosphaeria dieback resistance in *V. rupestris* are still elusive.

RNA-seq was applied in this study to reveal potential resistance mechanisms and genes associated with defense responses to *D. seriata* 98.1. Transcription factors (TFs), Gene Ontology (GO) terms and pathways related to the process of disease resistance were identified. The relationships between 53 TFs, three categories (biological process, cellular component and molecular function) of GO enrichment and 20 significantly enriched pathways and defense responses were analyzed. The data of differentially expressed genes (DEGs) enriched in subcellular components revealed the significant reaction of the anchored component of the plasma membrane, microtubule and cell wall to incubation with *D. seriata* 98.1. These results help to clarify the mechanism of responses in *V. rupestris* cells to Botryosphaeria dieback and will facilitate further studies to control GTD.

## 2. Materials and Methods 

### 2.1. Plant Material 

A suspension cell culture line of *V. rupestris* expressing GFP-*At*TUB6 was used in this study [21]. The cell line of *V. rupestris* expressing GFP-*At*TUB6 was kindly provided by Prof. Peter Nick (Karlsruhe Institute of Technology, Karlsruhe, Germany). Cells were cultivated in liquid medium containing 4.3 g L^−1^ Murashige and Skoog salts (Duchefa, Haarlem, The Netherlands), 30 g L^−1^ sucrose, 200 mg L^−1^ KH_2_PO_4_, 100 mg L^−1^ inositol, 1 mg L^−1^ thiamine and 0.2 mg L^−1^ 2,4-dichlorophenoxyacetic acid (2,4-D) which was adjusted to a final pH of 5.8 supplemented with 30 mg L^−1^ hygromycin B. Cells were subcultured weekly, inoculating 10 mL of stationary cells into 30 mL of fresh medium in 100 mL Erlenmeyer flasks. The cell suspensions were incubated in the dark at 26 °C on an orbital shaker (Shanghai ZhiCheng, Shanghai, China) at 150 rpm.

### 2.2. Incubation with Culture Filtrates of D. seriata 98.1 

The culture filtrates of *D. seriata* 98.1 (isolated from Pyrénées Orientales, France, kindly provided by Prof. Christophe Bertsch (Université de Haute-Alsace, Colmar, France)) were generated by cultivating 3 plugs of 2×1 cm of the strain grown on solid medium containing 37 g L^−1^ potato dextrose broth (PDB, Solarbio, Beijing, China) with 15 g L^−1^ agar powder (Solarbio, Beijing, China) for 1 week in 250 mL 20 g L^−1^ malt-medium (Bacto malt-extract, Solarbio, Beijing, China) in a 500 mL Erlenmeyer flask in the dark at 28 °C, 200 rpm for 21 days. Further, the *D. seriata* 98.1 culture was filtered by sterilized filter papers to remove the big fungi clusters and the liquid was harvested in an Erlenmeyer flask. After the liquid was vacuum filtered through a 0.22 μm filter, it was filled into sterilized 20 mL screw cap vials and stored at −20 °C.

An amount of 40 μL of culture filtrate of *D. seriata* 98.1 was added to the GFP-*At*TUB6-expressing suspension cells when subcultured. Sterile water (40 µL) was used in the mock-treated control. The cells treated in this way were incubated in the dark at 26 °C, 200 rpm, and were used 3 dpi for RNA extraction. Two biological replicates each comprising three technical replicates were conducted for each treatment.

### 2.3. RNA Extraction

The total RNA of the incubated cells was extracted with the RiboMinusTM Plant Kit for RNA-Seq (Invitrogen, Carlsbad, CA, USA) and digested by DNase I (Takara, Kyoto, Japan) subsequently according to the manufacturer’s instructions. The NanoDrop 2000 spectrophotometer (Thermo Scientific, Waltham, MA, USA) was used to determine the concentration and purity (260/280 and 260/230 ratios) of the total RNA.

### 2.4. Transcriptome Sequencing and Analyses

mRNA enrichment, mRNA fragment, cDNA synthesis, size selection, PCR amplification, quality control (QC) and sequencing were carried out with Total Genomics Solution provided by the company of HengChuang (TGS, Shenzhen, China) for preparing Illumina RNAseq libraries. All sequence data were filtered to remove adaptor sequences and low-quality sequences. The clean reads were aligned to the reference genome (https://www.ncbi.nlm.nih.gov/genome/401) by the comparison software HISAT v0.1.6. The fragments per kilo base per million reads sequenced (FPKM) measure was introduced to reflect the expression level [22]. The differentially expressed genes analysis was carried out by DESeq2 v1.4.5 [23]. Screening conditions of DEGs were difference multiples at least greater than 2 and a Q-value less than 0.01 at least. The functional annotation of DEGs was performed by GO enrichment (*p*-value < 0.05) [24]. The pathway enrichment of DEGs was analyzed by the Kyoto Encyclopedia of Genes and Genomes (KEGG, the major public pathway-related database). The RNA-seq data have been deposited to the online NCBI Sequence Reads Archive (SRA) database under the accession number PRJNA692532.

### 2.5. Real-Time Quantitative Reverse Transcription PCR

Complementary DNA (cDNA) libraries were synthesized from 1 μg total RNA by the PrimeScriptTM RT reagent Kit with gDNA Eraser (Takara, Kyoto, Japan). Subsequently, real-time quantitative reverse transcription polymerase chain reaction (qRT-PCR) was carried out with SYBR qPCR SuperMix Plus (Takara, Kyoto, Japan) on a CFX96TM Real-Time System (Bio-Rad, Hercules, CA, USA) on 12 genes selected among the DEGs that respond to *D. seriata* 98.1 incubation, in a total volume of 10 μL containing 5 μL SYBR qPCR SuperMix Plus, 0.2 μL each primer at 0.2 μM and 1 μL cDNA synthesized above. The thermal cycling consisted of a hold at 95 °C for 1 min, followed by 45 cycles of 95 °C for 20 s, 58 °C for 20 s and 72 °C for 30 s, and then a melting curve program at 65 to 95 °C raised gradually by 0.5 °C every 5 s. The expression of grapevine actin was amplified as an internal control. The relative expression levels compared to the mock-incubated control were calculated using the normalized expression method (2^−ΔΔCT^). The primers used in this experiment of each selected gene are listed in Supplementary Table S1. Standard errors of the mean values were generated by two biological replicates each comprising three technical replicates conducted for each gene.

### 2.6. Phenotyping of Cellular Responses to D. seriata 98.1 

The responses of the MTs to 50% (*v*/*v*), 2% (*v*/*v*) and 0.1% (*v*/*v*) culture filtrates of *D. seriata* 98.1 were observed at 20 min, 24 h and 3 dpi, respectively, and compared to a control with the corresponding amount of water. Aliquots of 20 μL suspension cells were placed on a slide and covered with a coverslip for microscopy to observe the MTs. All samples were investigated under an inverted fluorescence microscope (IX-73, Olympus, Tokyo, Japan) with a hypersensitive camera (Photometrics PRIME) and a U plan super apochromat objective 40X/0.95, WD 0.18 (spring, c.c.0.11–0.23). Images were then processed by the software installed including an optimized deconvolution algorithm. A plug-in program, “The macros”, developed for Image J (http://rsb.info.nih.gov/ij/) was used to quantify fluorescence skewness [25]. MTs images were skeletonized using the Image J menu “Plugins-kbi-Kbi_Filter2d (filter: lineFilters)-param (linemode: thinLine)”. 

Cell viability was quantified using 2% (*v*/*v*) culture filtrates of *D. seriata* 98.1 at 3 and 6 days after subcultivation/treatment by determining packed cell volume (PCV) according to Guan et al. [26]. To monitor changes in cell shape, the ratio of cell width over cell length was measured at 3 dpi with 2% (*v*/*v*) culture filtrates [26]. Each result presents 800–1000 cells scored from three independent experiments.

## 3. Results

### 3.1. Sequencing and Alignment 

**Figure 1:**
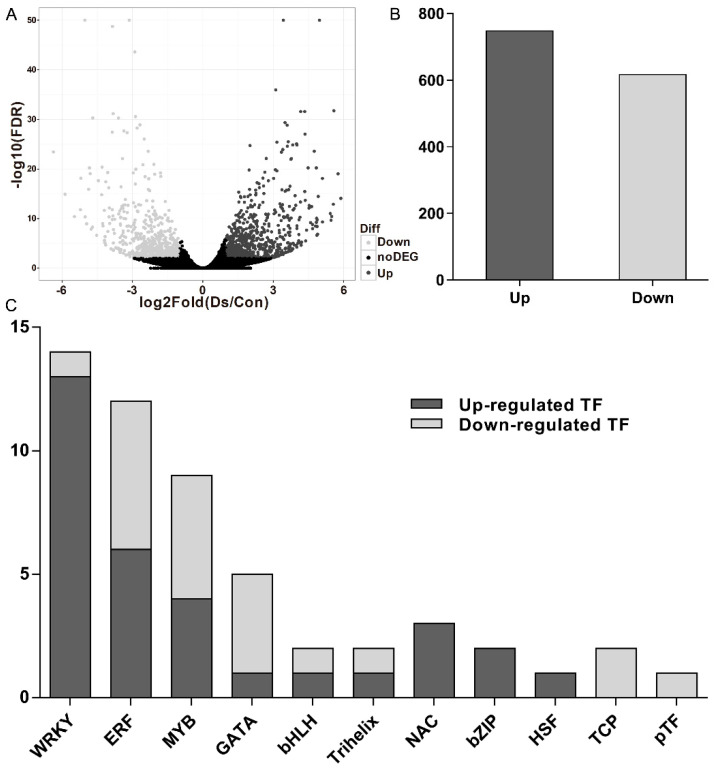
Differentially expressed genes (DEGs) (*p*-value < 0.05 and fold change > 2.0) in response to incubation with the culture filtrates of *D. seriata* 98.1 at 3 days post-inoculation (dpi) compared with the mock control. (**A**) Distribution of gene expression levels. The dark gray dots represent more prevalent transcripts in incubated cells than the control, while lower frequency is represented by light gray dots and transcripts showing no significant differences are represented by black dots. (**B**) The number of up-regulated and down-regulated genes in the incubated cells. (**C**) Differentially expressed transcription factor families of incubated cells.

Clean reads of DNA reversed from *V. rupestris* expressing GFP-*At*TUB6 incubated with *D. seriata* 98.1 and the mock-incubated control were generated by filtering to remove adaptor sequences and low-quality sequences. In addition, all clean reads were aligned to the reference genome and approximately 70% of them were mapped (Supplementary Table S2**)**. The Pearson’s correlation coefficient of the transcriptome profiles was 0.93, at least, between each biological replicate (Supplementary Table S3), indicating relatively high reproducibility of sequencing data. A total of 20,950 transcripts were detected (Figure 1A), and Supplementary Table S4 shows all the identified DEGs. A total of 1365 DEGs (748 were up-regulated and 617 were down-regulated, Figure 1B) were identified in *V. rupestris* expressing GFP-*At*TUB6 incubated with *D. seriata* 98.1 compared to the mock-incubated control.

### 3.2. Transcription Factors Analysis

Among the DEGs in the comparisons mentioned above, 53 TFs were obtained from the DEGs (Supplementary Table S5). These TFs were distributed in 11 families, and the dominant differentially expressed TFs were the members of the WRKY family (26.42%), followed by the ethylene-responsive factor (ERF) family (22.64%), the myeloblastosis (MYB) family (16.98%), the GATA family (9.43%), the NAC family (5.66%) and the basic helix-loop-helix (bHLH) family (3.77%) (Figure 1C).

### 3.3. GO Enrichment and KEGG Analysis of DEGs

Gene Ontology (GO) enrichment was carried out to functionally characterize the identified DEGs in *V. rupestris* expressing GFP-*At*TUB6 incubated with a culture filtrate of *D. seriata* 98.1 compared to the mock-incubated control. All the DEGs were classified into three categories including biological process, cellular component and molecular function. Compared to the mock-incubated control, 1098 GO terms were enriched (Supplementary Table S6). The extent of enrichment significance (Correct_*p*-Value) was calculated based on hypergeometric distribution and ranked in descending order in Supplementary Table S6.

Among them, the most significantly enriched biological process was defense response, followed by biosynthetic and oxidation–reduction processes. All the detected DEGs classified as defense response were up-regulated (Supplementary Table S7), for instance, *STS*, including *STS*, *STS1*, *STS2*, *STS5* and *STS6*, *PR* (pathogenesis-related protein), including *STH-2*, *PR10*, *PR10.3*, *PR10.4*, *PR10.8* and *PR10.9*, *MLO* (mildew resistance locus o), including *MLO7* and *MLO11*, *allergen Pru av 1* and *probable protein S-acyltransferase 7*. Some of the differently expressed *STSs* classified as defense response were enriched in biosynthetic process as well, while *STS3* (100249839), *STS2* (100855299), *STS5* (100264844) and *STS5-like* (100264173) were enriched in biosynthetic process, exclusively. Moreover, an aspartate aminotransferase and an alanine aminotransferase were enriched in biosynthetic process. Most of the DEGs that belonged to oxidation–reduction (such as *4 coumarate-CoA ligase*, *laccase*, *cytochrome dehydrogenase* and *cytochrome*) were up-regulated. In addition, the expression of *PAL*, including *PAL*, *PAL-like*, *PAL1-like* and *PAL G1*, was enriched in cinnamic acid biosynthetic process and L-phenylalanine catabolic process was enhanced. Noticeably, DEGs classified as defense response, the most significantly enriched term, were also enriched in response to biotic stimulus, except for *STS* and *SA7* (protein S-acyltransferase 7). On the contrary, DEGs of xyloglucan metabolic process (e.g., *xyloglucan endotransglucosylase/hydrolase 2*), cell wall biogenesis (e.g., *cellulose synthase-like protein D5*), microtubule-based movement (e.g., *tubulin β-4 chain*) and plant-type cell wall organization (e.g., *expansin-A14*) and most DEGs of cell wall organization (e.g., *tubulin β-1 chain*) were down-regulated. It is worth noticing that DEGs of DNA replication initiation (e.g., *DNA replication licensing factor MCM7*), regulation of DNA replication (e.g., *cyclin-D3-3*) and DNA methylation (e.g., *BEL1-like homeodomain protein 9*) were down-regulated, which were critical to cell growth and programmed cell death (Supplementary Table S7).

The anchored component of the plasma membrane was the most significant enriched cellular component, followed by the microtubule and cell wall (Supplementary Table S6). The DEGs enriched in anchored component of plasma membrane (e.g., *plasmodesmata callose-binding protein 5*) and the DEGs enriched in microtubule organization and movement (e.g., *tubulin α chain*) were down-regulated (Supplementary Table S7). Most of the DEGs enriched in cell wall, such as *expansin A4*, were down-regulated except for *pectinesterase 2* and *peroxidase 4*. The DEGs in kinesin complex and cytoskeletal part (e.g., *kinesin-like protein*) were down-regulated. In addition, the majority of DEGs enriched in plasmodesma, such as *expansion-B3*, were down-regulated, while the other seven DEGs (e.g., *hexose transporter*) were up-regulated (Supplementary Table S7).

**Figure 2:**
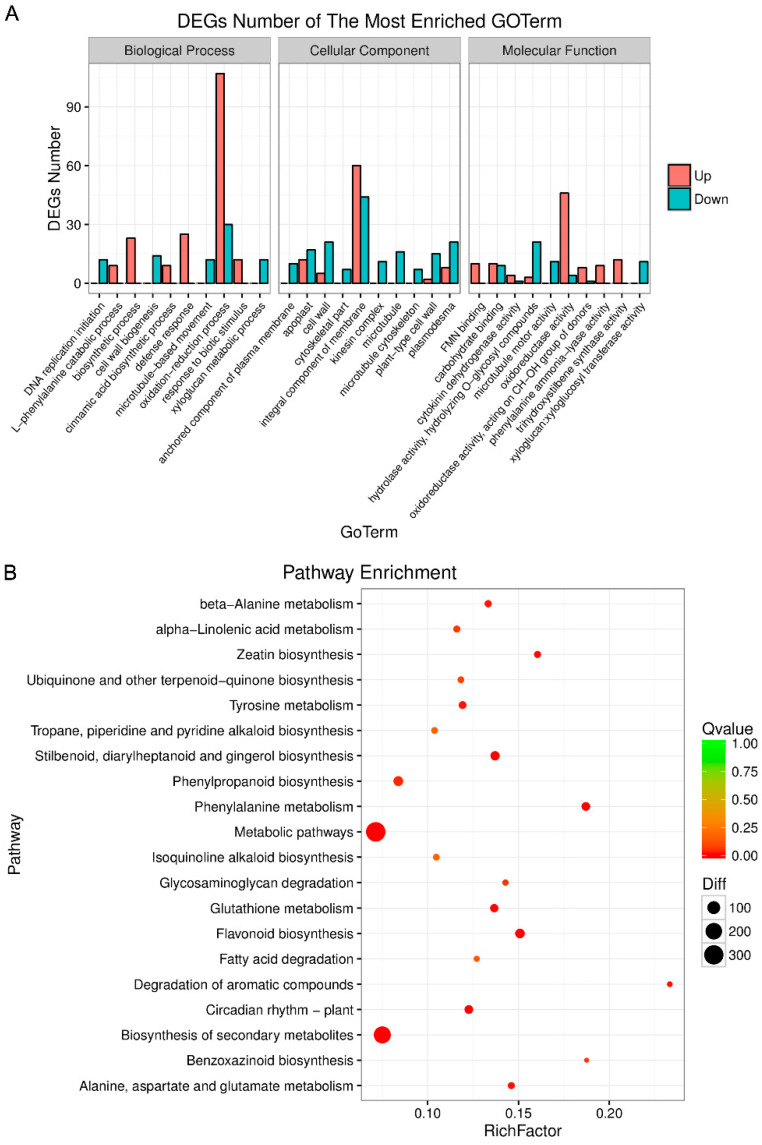
The enrichment of Gene Ontology (GO)- and Kyoto Encyclopedia of Genes and Genomes (KEGG)-based distribution of DEGs incubated with *D. seriata* 98.1. (**A**) The most significantly enriched biological process, cellular component and molecular function of GO enrichment (*p*-value < 0.05). (**B**) The DEGs enriched in different biological pathways were analyzed with the KEGG database. RichFactor represents the ratio of enriched DEGs to all genes in the corresponding pathway. Diff represents the number of DEGs enriched in the corresponding pathway. A lower Q-value represents a higher significant degree of enrichment.

Additionally, the most significantly enriched molecular function was trihydroxy stilbene synthase activity, followed by phenylalanine ammonia-lyase activity and carbohydrate binding (Supplementary Table S6). *STS* and *PAL* enriched in defense response and L-phenylalanine catabolic process of biological process, respectively, were up-regulated (Supplementary Table S7). Approximately 50% of the detected DEGs in carbohydrate binding were up-regulated; meanwhile, only few DEGs involved in hydrolase activity and hydrolyzing O-glycosyl compounds (*β-glucosidase 12 and cell wall apoplastic invertase*) were up-regulated. In addition, only four DEGs (e.g., *acyl-lipid (9-3)-desaturase*) were down-regulated in the oxidoreductase activity term. On the contrary, the DEGs enriched in xyloglucan (*xyloglucosyl transferase activity*), xyloglucan metabolic process, microtubule motor activity and microtubule-based movement were down-regulated (Supplementary Table S7). In biological process, cellular component and molecular function, the largest number of DEGs was found in oxidation–reduction process, integral component of membrane and oxidoreductase activity, respectively (Figure 2A).

Pathway enrichment analysis with the KEGG database was carried out with a *p*-value cutoff of <0.05 to understand the biological function of DEGs and to explore the biochemical pathways in which they were involved. The top 20 significantly enriched pathways are listed in Supplementary Table S8. The most significantly enriched pathway was flavonoid biosynthesis, followed by metabolic pathways, phenylalanine metabolism pathway, biosynthesis of secondary metabolites pathway and stilbenoid, diarylheptanoid and gingerol biosynthesis pathway. The circadian rhythm–plant pathway was related to environmental adaptation, while the other 19 significantly enriched pathways were related to metabolism. The largest amount of DEGs were enriched in metabolic pathways; the degradation of aromatic compounds pathway possessed the highest rich factor (calculated by DEGs in term/all genes in term in Supplementary Table S8) (Figure 2B). To be mentioned, 45 DEGs were up-regulated and 26 DEGs were down-regulated in the plant–pathogen interaction pathway (Supplementary Table S9).

### 3.4. DEGs in Enriched Pathways Related to Resistance Activated by D. seriata 98.1 Incubation

The most significantly enriched pathway, the flavonoid biosynthesis pathway, possessed 38 DEGs, of which only two genes were down-regulated (Supplementary Table S9). Among them, 21 genes were classified as STS (stilbene synthase), 2 as SNS (S-norcoclaurine synthase 1), 1 as TCMO (trans-cinnamate 4-monooxygenase-like), 1 as SOHCT (shikimate O-hydroxycinnamoyl transferase), 2 as SRG1 (encoding protein SRG1), 1 as DMS (encoding protein DOWNY MILDEW RESISTANCE 6), 1 as IFRL1 (isoflavone reductase-like protein 1), 1 as IFRHL (isoflavone reductase homolog-like), 1 as GHL (geraniol 8-hydroxylase-like), 1 as FS/FH (flavonol synthase/flavanone 3-hydroxylase), 1 as CYP (cytochrome P450 CYP73A100), 1 as CHI (chalcone isomerase), 1 as ANR (anthocyanidin reductase) and 1 as AT (uncharacterized acetyltransferase At3g50280-like). Conversely, one gene involved in PP (purine permease 1) and one in PLR (probable pinoresinol-lariciresinol reductase 3) were down-regulated.

There were 71 DEGs functioning in the plant–pathogen interaction pathway (Supplementary Table S9), which included 3 genes involved in CDPKs (calcium-dependent protein kinase, 1 up-regulated, 2 down-regulated), 2 in ERF (ethylene-responsive transcription factor, 1 up-regulated, 1 down-regulated), 13 in LRR (leucine-rich repeat related protein, 6 up-regulated, 7 down-regulated), 3 in LDP (lysM domain related protein, 2 up-regulated, 1 down-regulated), 5 in DRP (disease resistance protein, 2 up-regulated, 3 down-regulated), 15 in WRKY (WRKY transcription factor, 14 up-regulated, 1 down-regulated), 4 in MYB (MYB family transcription factor, 2 up-regulated, 2 down-regulated) and 2 in RP (receptor-like protein, 1 up-regulated, 1 down-regulated). Moreover, one gene in DFR (dihydrofolate reductase), one in CoAS (CoA synthase), one in BRIRK (Brassinosteroid insensitive 1-associated receptor kinase), two in CP (calmodulin protein), four in MRP (myb-related protein), two in RBOHP (respiratory burst oxidase homolog protein), one in SERK (somatic embryogenesis receptor kinase) and one in SF (splicing factor) were up-regulated. On the contrary, two genes involved in PR, two in CBP (calcium-binding protein), one in FKP (F-box/kelch-repeat protein), one in FP (formin-like protein), one in CNGC (protein CNGC15b) and one in SR (systemin receptor) were down-regulated.

A total of 48 DEGs were identified in the plant hormone signal transduction pathway (Supplementary Table S9), including 7 genes classified as regulating AUXIN (encoding auxin-induced/responsive protein, 1 up-regulated, 6 down-regulated), 3 involved in IAA regulation (encoding indole-3-acetic acid related protein, 2 up-regulated, 1 down-regulated), 3 involved in MYB (myb family transcription factor, 1 up-regulated, 2 down-regulated), 5 involved in LRR regulation (encoding leucine-rich repeat related protein, 3 up-regulated, 2 down-regulated), 2 involved in PHR1 regulation (encoding phosphate starvation response protein, 1 up-regulated, 1 down-regulated) and 2 involved in SLP regulation (encoding scarecrow-like protein, 1 up-regulated, 1 down-regulated). Moreover, one gene regulating ABA (abscisic acid receptor), one gene regulating BRIRK (Brassinosteroid insensitive 1-associated receptor kinase), two genes regulating bZIP (bZIP transcription factor), one gene regulating JA (jasmonic acid-amido synthetase), one gene regulating PSR (phytosulfokine receptor), one gene regulating CE (carboxylesterase), one gene regulating SERK (somatic embryogenesis receptor kinase), one gene regulating SP (sucrose-phosphatase) and one uncharacterized gene were up-regulated. In contrast, two genes classified as PR (encoding pathogenesis-related protein), two genes as CD (cyclin-D3), one gene as IRK (inactive receptor kinase), one gene as RPK (receptor protein kinase), seven genes as XEHP (encoding xyloglucan endotransglucosylase/hydrolase protein), one gene as SPBP (encoding squamosa promoter-binding-like protein) and one gene as bHLH (bHLH transcription factor) were down-regulated.

### 3.5. Real-Time Quantitative Reverse Transcription PCR Analysis

**Figure 3:**
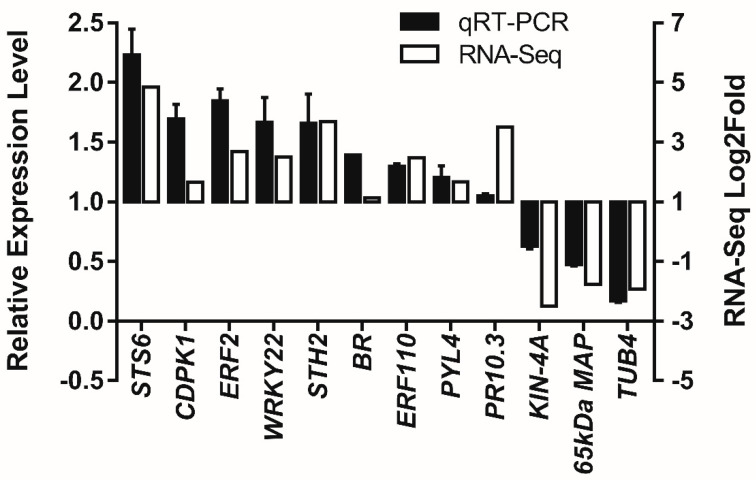
qRT-PCR analysis of relative expression levels of 12 selected DEGs in *V. rupestris* suspension cells incubated with *D. seriata* 98.1 and sterile water as the mock-incubated control. Actin was used as the reference gene. The left *Y*-axis represents the relative expression levels by qRT-PCR (black bar), while the right *Y*-axis represents the Log2Fold of each selected gene in RNA-seq results (white bar). Mean values and standard errors were obtained from two biological replicates.

To verify the results obtained by RNA-seq, the relative expression levels of 12 genes selected from DEGs were analyzed using qRT-PCR (Figure 3). Incubated cells and the mock control of suspension cells of *V. rupestris* expressing GFP-*At*TUB6 were analyzed by measuring the relative expression levels of these genes at 3 dpi incubated with the culture filtrates of *D. seriata* 98.1. The trends of four genes in the plant–pathogen interaction pathway, *CDPK1* (calcium-dependent protein kinase 1), *BR* (Brassinosteroid insensitive 1-associated receptor kinase 1), *ERF2* (ethylene-responsive transcription factor 2) and *WRKY22* (WRKY transcription factor 22), were confirmed to be in agreement with the fold changes of selected genes obtained from RNA-seq results (Figure 3). Similar patterns were observed not only for the genes related to plant hormone signal transduction including *PYL4* (abscisic acid receptor PYL4) and *BR*, but also for the genes enriched in the biological process defense response, including *STH-2* (encoding pathogenesis-related protein STH-2) and *STS6* (stilbene synthase 6). Moreover, *ERF110* (ethylene-responsive transcription factor ERF110) and *PR10* (pathogenesis-related protein 10) were also verified to be up-regulated. In contrast, one gene regulating microtubule cytoskeleton organization, *65 kDa MAP* (encoding 65-kDa microtubule-associated protein 3), and two genes in the biological process regulating microtubule-based movement, *TUB4* (tublin β-4 chain) and *KIN-4A* (kinasin-like protein KIN-4A), were down-regulated. The patterns between the results of qRT-PCR and those obtained from DEG data share high similarity, indicating reliable results of RNA-seq.

### 3.6. Phenotyping of Cellular Responses to D. seriata 98.1

**Figure 4:**
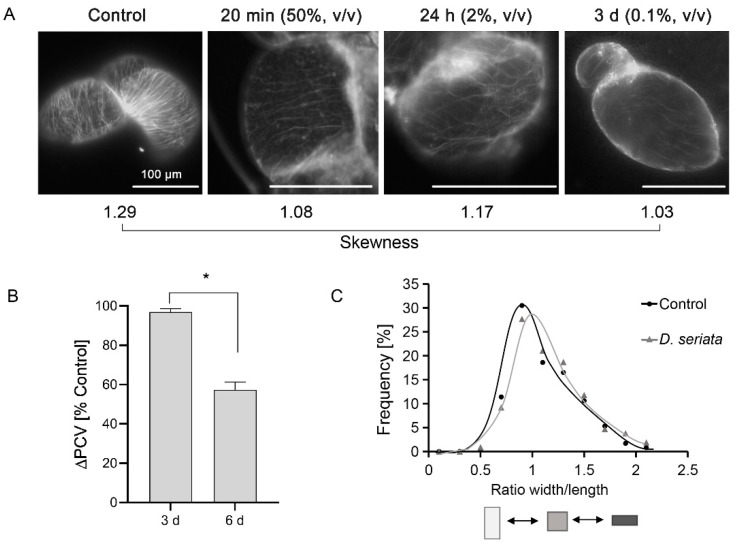
Phenotyping of cellular responses to *D. seriata* 98.1. (**A**) The response of the microtubules (MTs) to 50% (*v*/*v*), 2% (*v*/*v*) and 0.1% (*v*/*v*) culture filtrates of *D. seriata* 98.1 was observed in *V. rupestris* expressing GFP-*At*TUB6 at 20 min, 24 h and 3 days, respectively, with water as the mock control. Quantitative data for skewness are listed below the images. Observations are representative of at least three independent experimental series with a population of 20–30 individual cells for each treatment. (**B**) Packed cell volume as an indicator of culture growth at 3 and 6 days after incubation with 2% (*v*/*v*) culture filtrates of *D. seriata* 98.1, respectively. Data are mean ± standard errors from three biological replicates. Asterisks indicate significant differences evaluated using paired Student’s t test (* *p* < 0.05). (**C**) Frequency distributions for the ratio between cell width over cell length at 3 dpi with 2% (*v*/*v*) culture filtrates were measured from 800–1000 individual cells for each experiment to detect the changes in cell shape.

To put cell morphology in context with the transcriptome screening presented above, the MTs’ performance and cell phenotypes were further examined. Firstly, the MTs’ performance against 0.1% (*v*/*v*) culture filtrates of *D. seriata* 98.1 at 3 dpi was examined, where the MTs were depolymerized with a skewness value of 1.03 (skewness value is 1.29 in control cells) (Figure 4A). As the cytoskeleton participates in early signaling transduction in plant defense as a sensor and integrator [13], the remodeling of microtubules at earlier time points was interesting to observe. A compromised filtrate concentration has to be carefully considered with the incubation time (where MTs rearrangement is different between a prompt response with a higher concentration vs. a long-term response with a mild concentration). Images at 24 hpi (with culture filtrate of 2%, *v*/*v*) and 20 min post-incubation (with culture filtrate of 50%, *v*/*v*) are shown in Figure 4A. Such cytoskeletal responses can consequently alter cellular morphogenesis [27]. Packed cell volume (ΔPCV) was affected slightly by 2% (*v*/*v*) *D. seriata* 98.1 at 3 dpi; nevertheless, such influence was significantly increased at 6 dpi (Figure 4B). This leads to the hypothesis that the decreased ΔPCV was not due to the disrupted mitosis, but rather to the influence of cell expansion. The further experiments supported our hypothesis that the mitosis index showed no significant differences at 3 dpi (data not shown here) when it should be the most activated phase of mitosis in this cell line [21]. Since the organization of cortical microtubules can affect the axiality of cell expansion, the cell shapes were investigated at 3 dpi. Frequency distributions for the ratio between cell width over cell length were constructed (Figure 4C). The number of short-wide cells was increased after incubation with 2% (*v*/*v*) culture filtrates of *D. seriata* 98.1 at 3 dpi. 

## 4. Discussion

To study the defense responses of *V. rupestris* to Botryosphaeria dieback, a high-throughput DNA sequencing technology, RNA-seq, was utilized. After filtering the reads, all DEGs were aligned to the reference grapevine genome. In addition to finding out which TFs are possibly involved in the defense response in *V. rupestris* cells after incubation with culture filtrates of *D. seriata* 98.1, the function annotation and pathway enrichment of DEGs were analyzed by GO and KEGG, respectively.

Previous studies demonstrated that several types of TFs were involved in stress responses, and many TF genes are associated with enhanced tolerance to biotic/abiotic stresses [28]. In the present study, ERF, WRKY, MYB and bHLH were detected to respond to *D. seriata* 98.1 incubation. The roles of ERF transcription factors in response to stress encountered by plants have been reported [29]. Most of the ERFs are activators of biotic stress-responsive genes, whereas certain ERFs act as repressors. The majority of ERFs in the present study (*ERF2, ERF5, ERF11, ERF27* and *ERF110*) were up-regulated. The up-regulation of *ERF2* and *ERF11* coincided with the identified activators in plant defense [30,31]. Conversely, the down-regulated *ERF4* and *ERF5* have been reported as repressors [30,32]. The expression levels of *ERF2* and *ERF110* were verified by qRT-PCR (Figure 3). The results suggest they probably act as activators in plant resistance for grapevine, which has been rarely studied. The expression of *WRKY22* was verified as well (Figure 3). Corresponding with the trend of FPKM, the relative expression level of *WRKY22* was up-regulated. How *WRKY22* acts in the defense response of grapevine has not been studied, while reports on *Arabidopsis thaliana* revealed that the innate immunity triggered via *WRKY22* may protect against further pathogen infection [33]. *WRKY9, 14, 17, 26, 31, 65* and *72,* up-regulated in this study, have been verified to play important roles in responses to abiotic and biotic stresses [34,35,36]. On the contrary, a down-regulated *WRKY70* in the present study was considered as a vital node in the SA signaling pathway in plant defense responses [37].

According to the result of Ramirez-Suero et al. (2014), extracellular compounds produced by *D. seriata* 98.1 induced defense gene expression in *V. vinifera* cv. Chardonnay [12]. The research of Stempien et al. (2018) suggested that secreted proteins of *D. seriata* 98.1 induced a different defense gene expression pattern between the suspension cells of *V. vinifera* cv. Gewurztraminer and *V. rupestris* [4]. The GO terms and KEGG were performed to elucidate defense gene functional classifications and their secondary metabolic pathways in *V. rupestris* cells affected by culture filtrates of *D. seriata* 98.1 in this study. Up- and down-regulated DEGs were significantly enriched in biological process, cellular component and molecular function. According to the biological process of GO enrichment (Supplementary Table S7), STS (stilbene synthase), including *STS, STS1, STS2, STS3, STS4, STS5* and *STS6,* and *PAL* (phenylalanine ammonia-lyase), including *PAL, PAL1-like, PAL G1* and *PAL-like*, were up-regulated (Supplementary Table S7). These results from suspension plant cells/fungi culture filtrate incubation show a comparable pattern with that obtained from a one-year-old woody stem inoculated with mycelium [14]. Moreover, the expressions of *PR10, PR10.3, PR10.4, PR10.8* and *PR10.9* and *STH2* were identified as up-regulated in encoding secretion proteins related to the defense response. Expressions of *PR10.3* and *STH2* were further confirmed by qRT-PCR (Figure 3). Rather than the above PR genes expressed in *V. rupestris*, *PR6* and *Chit4c* were reported to be expressed in *V. vinifera* incubated with extracellular compounds of *D. seriata* [12]. In this publication, it indicates that the expression levels of these two defense genes are significantly higher at 3 dpi than other tested time points, which drove us to exam the gene expression level at the same time point in *V. rupestris* cells as a resistant counterpart to *V. vinifera* cv. Chardonnay. So far, there have been no studies on how *STH2* responds to pathogens in grapevine. Although *STH2* was rapidly activated in potato infected with spores of *Phytophthora infestans*, a genus of plant-damaging oomycetes [38], *STH2* and *PR10* were, for the first time, reported in response to grapevine fungus disease in this study. In addition, most (78%) of the DEGs (137) functioning in oxidation–reduction in biological process were up-regulated (Supplementary Table S7). A similar process was found in *D. seriata-*infected olive trees [39]. On the other hand, DEGs in xyloglucan metabolic process, cell wall biogenesis, microtubule-based movement and plant-type cell wall organization and most DEGs in cell wall organization were down-regulated; additionally, DEGs in DNA replication initiation, regulation of DNA replication and DNA methylation were down-regulated, which were critical to cell growth and programmed cell death. The down-regulation of these genes related to cell structure and replication probably enables cells to allocate more energy to resistance in the presence of a pathogen [40]. 

Moreover, a few genes encoding inducible defense-related compounds were identified in biological process of GO enrichment. *VST1* has been studied by fusing it to a fungal-inducible promoter in grapevine [41]. Knockdown of *MLO7* in combination with *VvMLO6* and *VvMLO11* was reported to enhance resistance to powdery mildew in *V. vinifera* [42]. *VST1, MLO7* and *MLO11* were found, for the first time, in this study to be up-regulated significantly in *V. rupestris* incubated with *D. seriata* 98.1. Stilbenoids representing major antimicrobial compounds in grapevine could be elicited by fungal infection [43]. In this study, the transcriptome analysis showed that all 21 identified *STS* genes were up-regulated. The expression of *STS* could produce stilbene phytoalexins whose accumulation suggests them as the most frequently observed anti-pathogen metabolites, and their defense mechanisms were well characterized [44]. The qRT-PCR result of *STS6* gave an example to verify the up-regulation of the genes in the stilbenoid biosynthesis pathway (Figure 3).

GO enrichment analysis provided information of the DEGs in cellular component and molecular function (Supplementary Table S7). In terms of cellular component, the down-regulation of DEGs enriched in plasma membrane and microtubule revealed their roles in response to fungal filtrates. The DEG related to microtubule-based movement was mainly the kinesin-liked protein. Those genes encoding tubulin α chain, tubulin β-1 chain and tubulin β-2 chain were enriched in microtubule of cellular component. Likewise, the down-regulation of DEGs in cell wall, e.g., *expansin A4*, limited the maintenance of cell rigidity, as well as regulating the growth and division of cells. The majority of DEGs enriched in the category of plasmodesma were down-regulated, which probably functioned in dispelling plasmodesmata under the fungal filtrates incubation. In terms of molecular function, the accumulations of STS and PAL in trihydroxy stilbene synthase activity and phenylalanine ammonia-lyase activity, respectively, were enriched in biological process as well. Reduced xyloglucosyl transferase activity indicated that the processes of formation and enlargement of the cell wall were blocked partially. The down-regulation of DEGs enriched in microtubule motor activity revealed that the contributions of kinesin-like protein on microtubule-based movement were inhibited.

**Figure 5:**
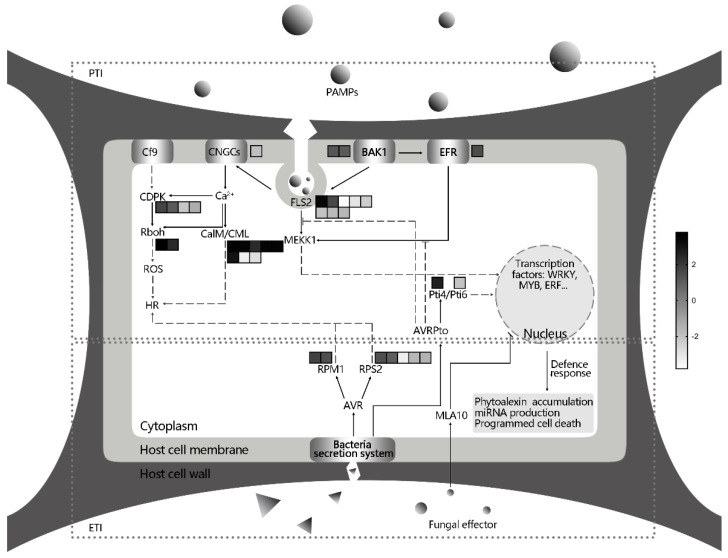
The patterns of pathogen-associated molecular patterns (PAMPs)-triggered immunity (PTI) and effector-triggered immunity (ETI) in *V. rupestris* cells after *D. seriata* 98.1 incubation. The squares represent the transcriptional changes of DEGs. The fold change is displayed as illustrated in the color bar on the right (black is up-regulated and white is down-regulated).

The innate immunity in plants is composed of two layers: the pathogen-associated molecular patterns (PAMPs)-triggered immunity (PTI) and effector-triggered immunity (ETI) [45]. At the level of PTI, receptor protein kinases (RPKs) on the plasma membrane represent the main pattern recognition receptors (PRRs) perceiving diverse PAMPs [46]. In *V. rupestris* cells incubated with *D. seriata* 98.1, three RPKs, including BAK1, FLS2 and EFR, were identified. As a central regulator of the PTI level, BAK1 is the target of several pathogen virulence effector molecules. Rather than influencing elicitor perception directly, it could rapidly form a complex with FLS2, the receptor for the bacterial flagellin, after elicitation [47]. The up-regulation of these two RPKs suggests that a complex may have formed after *D. seriata* 98.1 incubation in *V. rupestris* cells. In addition, this complex involved in endocytotic recycling of plant PAMP receptors is often necessary for signaling from intracellular compartments [48]. The EFR allows the plant cells to recognize and bind to bacterial EF-Tu and prevent genetic transformation and protein synthesis in the pathogen [49]. In the present study, the expression of *EFR* was up-regulated after incubation with culture filtrates of *D. seriata* 98.1. Involved in several cascades, these RPKs led to the activation of the WRKY transcription factors such as *WRKY33, WRKY29* and *WRKY22,* which induced the expression of defense-related genes (Figure 5). In addition, the transduction of the calcium signal through the cyclic nucleotide gated channel (CNGC) affected Rboh and CaM/CML, giving rise to the hypersensitive response (HR) (Figure 5) and cell wall reinforcement [50]. Moreover, the genes encoding CDPKs [51], WRKY [37], ERF [29], LRR [52], MYB [53] and PR [14] have also been reported to play resistant roles in biotic stress responses.

At the ETI level, the avirulent (Avr) protein induced a HR via a bacterial secretion system [31]. A fungal effector, AvrA10, activated the expression of *WRKY2* which triggered the downstream defense-related gene expression and programmed cell death in *V. pseudoreticulata* to powdery mildew [40]. In this study, five genes encoding disease resistance proteins (104881039 and 109121518 were up-regulated; 100855173, 104877588 and 104881511 were down-regulated) and one uncharacterized gene (100261821) were differentially expressed. Among them, two R-proteins (RPM1 and RPS2) were encoded. 

Plant hormones such as SA, JA, GA, ABA, auxin and brassinosteroids (BR) play key roles in the complex signaling cascades of defense responses [17,21,54,55]. Our previous findings showed that JA induced bundling of microtubules and auxin can revert such JA-induced bundling in *V. rupestris* suspension cells [21]. Fung et al. (2008) [54] showed that ABA is possibly involved in the inhibition of SA in grapevine resistance to *E. necator*, a causal agent of powdery mildew. However, the expression patterns of ABA and BR resistance to *D. seriata* 98.1 in this study were different from the resistance to *E. necator*. BR-associated receptor kinase 1 was up-regulated; meanwhile, no DEG related to ABA has been found yet (Supplementary Table S9). The concentrations of ABA and the glucose esters of ABA (ABA-GE) were increased after Eutypa dieback infection, which accumulated as the symptom became more serious. This may indicate the perturbation of ABA-GE translation or the synthesis of ABA [56]. SA was required for TGA1 (a transcription factor) to interact with NPR1 (a disease resistance protein) in plant cells [57]. Where some DRPs may serve as cofactors conferring redox regulation of DNA binding activity to TGA2.2 [58,59] which was identified in this study as a transcription factor (Supplementary Table S9).

The reactions of subcellular components to pathogens have been reported in plant cells. Amborabé et al. (2001) [15] found the plasmalemma is an essential target of a toxin produced by *Eutypa lata*, the causal agent of Eutypa dieback. Via plasmodesmata, SA signaling components regulate cellular connectivity in plant innate immunity [16]. In addition, dynamic reorganization of the cytoskeleton against fungal penetration attempts is a basic and prompt response. Not only playing important roles in penetration resistance by the polarization of defense-related reactions [19], actin filaments and microtubules are involved in the expression of hypersensitive reactions [60,61] and elicitor-induced signal transduction [62]. According to the cellular components of GO enrichment in this study, the expression of DEGs enriched in anchored component of plasma membrane, microtubule and cytoskeletal were all down-regulated, and most of the DEGs in plasmodesma were as well. As both *NACK2* and *65-kDa MAP* were down-regulated, the DEGs associated with cytoskeleton suggested that the formation of microtubules and the following regulation of cell development declined after incubation with *D. seriata* 98.1. The qRT-PCR results of *TUB4*, *KIN-4A* and *65kDa MAP* further verify the down-regulation of genes associated with microtubules (Figure 3). The rearrangements of MTs were examined at 3 dpi with a relatively mild culture filtrate concentration to provide the impression on cell morphology at the same timepoint and concentration when RNA-seq was conducted. Since the MTs’ responses to biotic/abiotic stresses are prompt, the images at 20 min and 24 h post-incubation with higher concentrations are presented in Figure 4A. Our results show that *D. seriata* 98.1 induced short-wide cells in the suspension cell line at 3 dpi. This abnormal phenotype was due to the depolymerization of MTs, which affected the axial expansion of cells. The long-term impact on ΔPCV at 6 dpi was caused by the individual reduced cell volume rather than the destroyed mitosis (Figure 4B, C) of the cell colony.

This research provides a comprehensive analysis of DEGs that respond to *D. seriata* 98.1. A total of 748 up-regulated and 617 down-regulated DEGs were identified. The result of RNA-seq was verified by qRT-PCR. Furthermore, transcription factors, GO terms and pathways related to defense responses were analyzed. Our results suggest that some important DEGs and transcription factors are involved in innate immunity, plant hormones and cell subcellular components, which play key roles in disease resistance. 

## Data Availability

The RNA-seq data has been deposited to the online NCBI Sequence Reads Archive (SRA) database under the accession number PRJNA692532.

## Supplementary Materials

The following are available online at https://www.mdpi.com/2073-4425/12/2/179/s1, Table S1: Primers for qPCR, Table S2: Reads sequenced and mapped to reference genome, Table S3: The pearson correlation coefficient, Table S4: List of DEGs identified, Table S5: Differentially expressed transcription factors, Table S6: GO enrichment, Table S7: DEGs list of significantly enriched GO terms, Table S8: Pathway Enrichment Analysis, Table S9: DEGs in enriched pathways related to resistance
